# Active Hexose Correlated Compound Activates Immune Function to Decrease *Chlamydia trachomatis* Shedding in a Murine Stress Model

**DOI:** 10.23937/2572-3278.1510006

**Published:** 2015-09-15

**Authors:** Tesfaye Belay, Chih-lung Fu, Anthony Woart

**Affiliations:** 1 School of Arts and Sciences, Bluefield State College, Bluefield, WV 24701, USA; 2 Current Address is the National Institute of Allergy and Infectious Diseases, NIH, Bethesda, MD 20892, USA

**Keywords:** Cold-induced stress, Chlamydia, active hexose correlated compound

## Abstract

A cold-induced stress mouse model for investigating chlamydia genital infection and immune response analysis was established in our laboratory. Previous results showed that cold-induced stress results in suppression of the immune response and increased intensity of chlamydia genital infection in the mouse model. The purpose of the present study was to evaluate the potential therapeutic value of active hexose correlated compound (AHCC) against chlamydia genital infection in mice. AHCC is an extract of mushroom commonly used as a dietary supplement is known to boost the immune system. Mice were infected intravaginally with *Chlamydia trachomatis* after a 24-day cold-stress application. Oral administration of AHCC to stressed or non-stressed mice was carried out seven days before infection and during the course of infection along with cervicovaginal swabbing. Cytokine production by peritoneal and splenic T cells isolated from AHCC-fed stressed mice and non-stressed mice was measured ELISA. Splenic T cells from both animal groups were co-cultured with mouse monocyte J774.2 cell line or cultured by addition of supernatants of AHCC-treated J774.2 cell line for 24 hours. Infection studies showed that AHCC-feeding compared to phosphate buffered saline (PBS)-feeding to stressed mice resulted in reduced *Chlamydia trachomatis* shedding from the genital tract. Levels of tumor necrosis factor-alpha (TNF-α) and interleukin 6 (IL-6) were significantly increased in stressed mice receiving AHCC compared to stressed mice receiving PBS. Production of interferon gamma (IFN-γ) and interleukin 2 (IL-2) in the AHCC group was significantly high compared to production in PBS-fed group. Splenic T cells from stressed and non-stressed cultured with supernatants of AHCC-treated J774.2 cell line resulted in significantly increased TNF-α or IFN-γ production. Results obtained in this study show that AHCC improves the function of immune cells as indicated by the restoration of levels of cytokines production that were suppressed under cold induced-stress conditions. This is the first report showing that oral administration of AHCC enhances the function of the immune system, which could result in increased resistance of the host to chlamydia genital infection.

## Introduction

Epidemiologic data from the World Health Organization (WHO) and Centers for Disease Control and Prevention (CDC) have revealed that *Chlamydia trachomatis* genital infection is a serious public-health problem, with more than 90 million new cases occurring annually worldwide and 4 million in the USA alone [1–3]. Chlamydia genital infection in the United States and other countries disproportionately affects populations of low socio economic status [4,5]. It is well known that stress has a significant impact in public health, and studies find that stress is generally greater in populations of lower socioeconomic status [6]. Although numerous biological, epidemiological and clinical studies of chlamydia genital infection have been undertaken, the relationship of stress in the pathogenesis of chlamydia genital disease and its influence on the immune response against the disease remains unknown.

Information on the impact of stress-induced changes on immune response and risk of infectious diseases is growing. Different approaches of animal exposure to cold water have been widely used to study the effects of stress on resistance to infection [7–9]. In this stressing method, rodents are exposed to 4°C water and forced exercise of swimming that invokes a complex paradigm of stressors, including anxiety and hypothermia [9]. Application of cold water as stressor in animal models including mice has resulted in changes in levels of immunological parameters, corticosteroids, and catecholamines. Release of stress hormones such as corticosterone and norepinephrine have been shown to inhibit immune responses and decrease resistance to bacterial infections [10–12]. For instance, mice and rats exposed to cold water stress displayed decreased numbers of immune cells and a decreased capacity to secrete certain cytokines [8,13].

Studies in human subjects have showed that natural substances that are nontoxic to cells can effectively treat disease and rejuvenate immune responses [14–16]. In recent years, AHCC has received special attention as a food supplement and alternative medicine in human subjects [17–19] and animal models [20–22]. AHCC is an extract prepared from co-cultured mycelia of several species of edible *Basidiomycete* mushrooms [14,17,23]. AHCC has been made commercially available by Amino Up Chemical Company and is most common in Japan and the US as a nutritional supplement taken orally. Recent studies have shown that AHCC promotes T helper (Th) 17 and Th1 cell responses by inducing interleukin-1beta (IL-1β) production from monocytes in humans [24]. Other studies suggest that AHCC enhances CD4^+^ and CD8^+^ T cell immune responses in healthy elderly persons [25].

Animal infection studies have shown that AHCC supplementation to tumor-bearing mice [26] resulted in an improvement of the immune response to acute influenza infection in C57BL/6 mice [27,28] and endocrine disturbance [29]. Similarly, low viremia levels, higher survival rate, and protective immunity was observed in West Nile virus infected mice that received AHCC (600 mg/kg) every other day for 1 week before and at day 1 and 3 post-infection [30]. Our research group has previously shown that AHCC supplementation leads to activation and induction of the immune response, subsequently leading to an effective rapid clearance of bacteria from infected mice [31]. Furthermore, researchers have shown that AHCC is anti-inflammatory and could be useful as a prebiotic in the design of functional foods for inflammatory bowel disease patients [32,33]. Our previous studies showed that hindlimb-unloaded mice that received AHCC for 1 week before and during the infection period overcame the effects of hindlimb unloading and showed improved survival, approaching levels seen in normally-housed mice [34]. AHCC supplementation has decreased susceptibility of a host of a variety of infectious agents, including influenza virus, avian influenza virus, *Klebsiella pneumonia, Candida albicans, Pseudomonas aeruginosa*, and methicillin-resistant *Staphylococcus aureus* [35]. Supplementation of AHCC has also shown to suppress the internucleosomal DNA fragmentation in the thymus induced by dexamethasone as demonstrated by flow cytometry analysis [36].

The molecular mechanisms involved in the enhancement of the immune system by AHCC under normal and cold-induced stress conditions remain unclear. However, it is probable that the high content of carbohydrates present in AHCC plays a role in the regulation of the immune system. The structural components of AHCC contain a mixture amino acids, minerals, and polysaccharides (with 74% of oligosaccharides and 20% of the -1, 4-glucan type) [37]. The acetylated forms of the -1, 4-glucans are believed to be the molecules responsible for the biological activities of AHCC to restore immune function [16].

AHCC has been shown to have an enhancing effect on the immune system of humans [16,23,32] including an increase of natural killer cell activity and IL-12 production [24]. In recent years, there has been a growing body of evidence that non-protein antigens such as glycolipids can be recognized by T cells and that antigen presentation requires the binding of peptides to the CD1 molecule [37]. Experiments using model T cell epitopes also have demonstrated that carbohydrates can modulate T cell responses in a variety of ways [38–40]. Several mechanistic studies have shown that beta-glycan and a lectin-like receptor, Dectin-1, have a major role in the stimulatory effect of the immune system [41–43], but it is not well defined.

The objective of this study was (i) to test whether feeding AHCC to cold-induced stressed mice decreases Chlamydia shedding from genital tract; (ii) explore the mechanisms involved in AHCC-enhanced resistance to *Chlamydia trachomatis* genital infection in a cold-induced stress model. The hypothesis tested was that oral administration of AHCC restores the function of the immune system of cold-induced stressed mice leading to decreased intensity of Chlamydia genital infection.

## Materials and Methods

### *Chlamydia trachomatis* stock culture and McCoy cells

*Chlamydia trachomatis* agent of mouse pneumonitis (MoPn) biovar (strain Nigg) and McCoy mouse fibroblast cell line were kindly provided by Dr. Joseph Igietseme, Morehouse School of Medicine; Atlanta, GA.

### Animals

Specific pathogen-free female 6–7-wk-old BALB/c mice, each weighing 16 to 18 g, purchased from Hilltop Animal Lab Inc. (Scottsdale, PA) were used throughout the study. Animals were housed in a quiet, isolated room with controlled temperature and light cycle, and given *ad libitum* food and water. Experimental procedures were commenced after 1 week of acclimation. All experimental manipulations were approved by the Bluefield State College Institutional Animal Care and Use Committee.

### Weight gain determination

Mice were weighed upon arrival and at the start of AHCC and PBS feeding. At least 10 mice each of stressed and non-stressed groups were weighed daily during the last seven days of the stressing and feeding period. The weight of stressed mice was compared to the weight of non-stressed mice. Mice were sacrificed by CO_2_ inhalation followed by cervical dislocation and the spleens were harvested and weighed. A statistical comparison of weights of spleens of stressed and non-stressed mice was performed.

### Cold water stress protocol

Cold-induced *s*tress application has been established in our lab [13]. Stress was applied by placing mice in a packet filled with 4 cm of cold water (1–4°C) for 5 minutes daily for 24 days. The water level was deep enough to cover the animals’ backs while swimming in water. At the end of each stressing period, mice were dried with towels to avoid hypothermia. Non-stressed mice were kept at room temperature without treatment with cold water.

### Course of *Chlamydia trachomatis* genital infection

All AHCC or PBS-fed and non-fed mice received 2.5 mg of progesterone (Depo-Provera) in 100 µL of PBS subcutaneously 7 days before infection to synchronize estrous cycle and increase mouse susceptibility to Chlamydia genital infection. Groups of 5 or 6 stressed or non-stressed mice were inoculated intravaginally with 10^3^ IFU of *C. trachomatis* in a volume of 30 µL of PBS while under Ketamine-Xylazine-induced anesthesia. The course of infection was monitored by cervico-vaginal swabbing at 3-day intervals for the first 40 days of the primary course of infection. The total of infection in the cervico-vaginal vault was measured by the isolation of *Chlamydia trachomatis* from the swabs in tissue culture followed by immunofluorescence staining and enumeration of inclusions, as previously described [13]. Briefly, vaginal swabs were cultured in monolayers of McCoy cells and stained with fluorescein-labeled specific antibodies against *C. trachomatis. Chlamydia* inclusion bodies were enumerated under fluorescence microscope for inclusion forming units per milliliter (IFU/mL) determination following the standard procedures previously described [13].

### Administration of active hexose correlated compound (AHCC)

Active hexose correlated compound (AHCC) was provided by the Amino Up Chemical. Co. Ltd. (Sapporo, Japan). AHCC in powder was weighed and dissolved in PBS and administered to each mouse by a plastic feeding tube (20 ga × 30 mm) following the manufacturer’s recommendation (Instech Laboratories Inc., Plymouth, PA). Dose was adjusted to a 600 mg/kg concentration for oral administration of 300 µL daily starting seven days before the end of the 24-day stressing period and during the course of infection and swabbing. To ensure that the observed changes were not due to the gavage procedure, a group of mice received the excipient (PBS) during the course of infection.

### Detection of *Chlamydia trachomatis* using Anti-chlamydia antibody staining method

McCoy cells were seeded into a 96-well plate and grown to a 90% confluence. Supernatant was aspirated and replaced with a volume of 200 µL of supernatant collected from the genital tract swabs and centrifuged for 1 hour at 3000 rpm to attach *Chlamydia trachomatis* inclusion bodies to the monolayers. This step was followed by incubation at 37°C, 5% carbon dioxide for 2 hours. The old supernatant was removed and replaced with a volume of 200 µL of complete cycloheximide medium into each well after which the plate was incubated at 37°C, 5% carbon dioxide for 32 hours. The supernatant was removed and replaced with 200 µL of methanol and the plate was placed in the refrigerator for overnight to preserve the cells. The methanol was removed and the plate was washed twice before 30 µL of anti-chlamydia antibody was added to each well. The plate was held at room temperature covered in aluminium foil for 1 hour. After a final wash with PBS the plate was left overnight to dry completely and glycerol was added to each well for preservation. Inclusion bodies from at least 5 wells of each treatment group (10–20 fields/well) were counted under fluorescence microscope and IFU/mL was determined as previously described [13].

### Isolation and proliferation of splenic and peritoneal cells

#### Peritoneal cells

Mice were injected with 1 mL of sterile 3% thioglycolate into the peritoneal cavity four days prior to cell harvesting. Cells from the different groups of mice were collected and placed in complete RPMI-1640 medium (Sigma, St. Louis, MO) supplemented with 10% fetal bovine serum (Sigma), 1% penicillin/streptomycin (Sigma), and 0.1% gentamicin (Sigma). After sacrifice, peritoneal cells were collected by lavage with 7 mL of sterile RPMI-1640 tissue culture media. Cells were washed in complete RPMI-1640.

#### Splenic T cells

The spleen was removed from each mouse, mashed aseptically and pressed through a cell strainer (Becton Dickinson, Franklin Lakes, NJ) for single cell suspension preparation. Erythrocytes were removed from the cell suspension by treatment with lysis buffer (0.83% NH_4_Cl in 0.1 nM Na_2_EDTA and 1 M KHCO_3_). T cells from spleen lysates were enriched by passing through nylon wool for one hour at 37°C and washing by centrifugation of eluent from column. Cell viability was determined by use of Trypan Blue (Cambrex Bio Science, Walkersville, MD). Both cell preparations were plated in quadruplicate at a density of 5 × 10^5^ cells per well in a 96-well flat bottom tissue culture plate (Corning Inc., Corning, NY). Spleen cells were stimulated with 2.5 µg/mL of concanavalin A (Con-A) and peritoneal cells were stimulated with 10 µg/mL of LPS. Plates were placed in a water-jacketed incubator at 37°C in 5% CO_2_. Supernatants were collected after 72 hours of proliferation for detection of cytokine production

### Cytokine measurement by enzyme-linked immunosorbent assay (ELISA)

Collection of supernatants from splenic cell and peritoneal cell proliferation cultures were used to determine cytokine production by ELISA. The concentrations of cytokines of IL-6, TNF-α, IL-2, and IFN-γ were determined by using the Douset ELISA system method according to manufacturer’s instructions (R&D systems, Minneapolis, MN).

### Immune cell isolation and proliferation experiments using dynabead kits

Harvesting of splenic cells was performed using a dynabead kit of Life Technologies (Carlsbad, CA) following the manufacturer’s instruction. Cells were allowed to proliferate for 72 hours, in presence or absence concanavalin A (ConA) and/or norepinephrine (NE) after which culture supernatants were collected for ELISA analysis.

### Antigen presenting cell (apc) isolation

Immunologically naive mice (non-stressed and non-infected) were used to isolate APCs by treating with mitomycin C. Total T cells from the non-stressed and non-infected group were set aside before applying the dynabead kit for T-cell isolation for treatment with mitomycin C. The isolated APCs were then applied to certain treatment groups to induce an *in vitro* proliferation in similar physiological conditions.

### Mouse monocyte cell culturing for IL-1β production

AHCC was dissolved in PBS, passed through 0.22 µm filters and used immediately. Mouse macrophage cell line (J774.2) was cultured in complete DMEM at 37°C, 5% CO_2_ for 24 hours in the presence of the filtered AHCC (at 100 µg/mL) or lipopolysaccharide (LPS, 10 mg/mL) or PBS (control). Culture supernatants were collected and stored at −20°C for ELISA and use in further experimentation of immune cell stimulation.

### Immune cell culturing with J744.2 cell line or AHCC-treated J744.2 culture supernatant

Splenic T cells harvested from stressed and non-stressed mice were co-cultured in the presence or absence of J774.2, or culture supernatant of AHCC-treated monocyte (J774.2). The same cultures were tested in the presence/absence of LPS, Con A, or norepinephrine (NE). The concentrations of cytokines produced in culture supernatants of T-cells cultured for 72 h were determined by using an ELISA kit (Invitrogen) following the manufacturer’s instructions.

### Statistical analysis

Groups of five non-stressed and stressed mice in each treatment were used in each study to allow for statistical analysis. Experiments were repeated at least twice using the same experimental conditions. For each cytokine measured, ANOVA was first used across all groups to detect any statistically significant differences, followed by the student’s t test to compare individual treatment groups for statistical significance (p ≤ 0.05).

## Results

### Oral administration of AHCC resulted in relative body weight gain in stressed mice

Previous and present studies have shown that stressed mice have lost more weight than non-stressed mice. In the present study, the restoration of body or spleen weight loss of AHCC-fed and PBS-fed stressed mice was compared. As expected, stressed mice suffered loss of weight before feeding, however, improvement in body weight gain was shown in AHCC-fed mice (21.6% gain) compared to PBS-fed mice (19.4% gain) (Table 1). The recovery in body weight was further reflected by a slight gain in spleen weight of AHCC-fed compared to the PBS-feed mice, suggesting that AHCC restores body and spleen weight of stressed mice.

### AHCC enhances resistance to Chlamydia trachomatis genital infection in stressed mice

Using the cold water-induced stress model, we investigated the effect of AHCC feeding on the intensity and the course of a primary genital *Chlamydia trachomatis* infection in female mice. Stressed and non-stressed mice orally received 300 mg/kg of AHCC per day for 7 days prior to infection with *Chlamydia trachomatis*. At day 3, stressed mice fed with AHCC showed a lower intensity of infection than PBS-fed mice, but no significant statistical difference between AHCC-fed and PBS-fed groups was measured. As shown on Table 2, a significant reduction in the shedding of *Chlamydia trachomatis* from the genital tract of AHCC-fed mice on day 18 after infection was observed (p ≤ 0.05). As expected all stressed and non-stressed mice were able to clear the primary infection by day 40 after *Chlamydia trachomatis* inoculation.

### AHCC restores the function of immune cells in a mouse stress model

**Figure 1:**
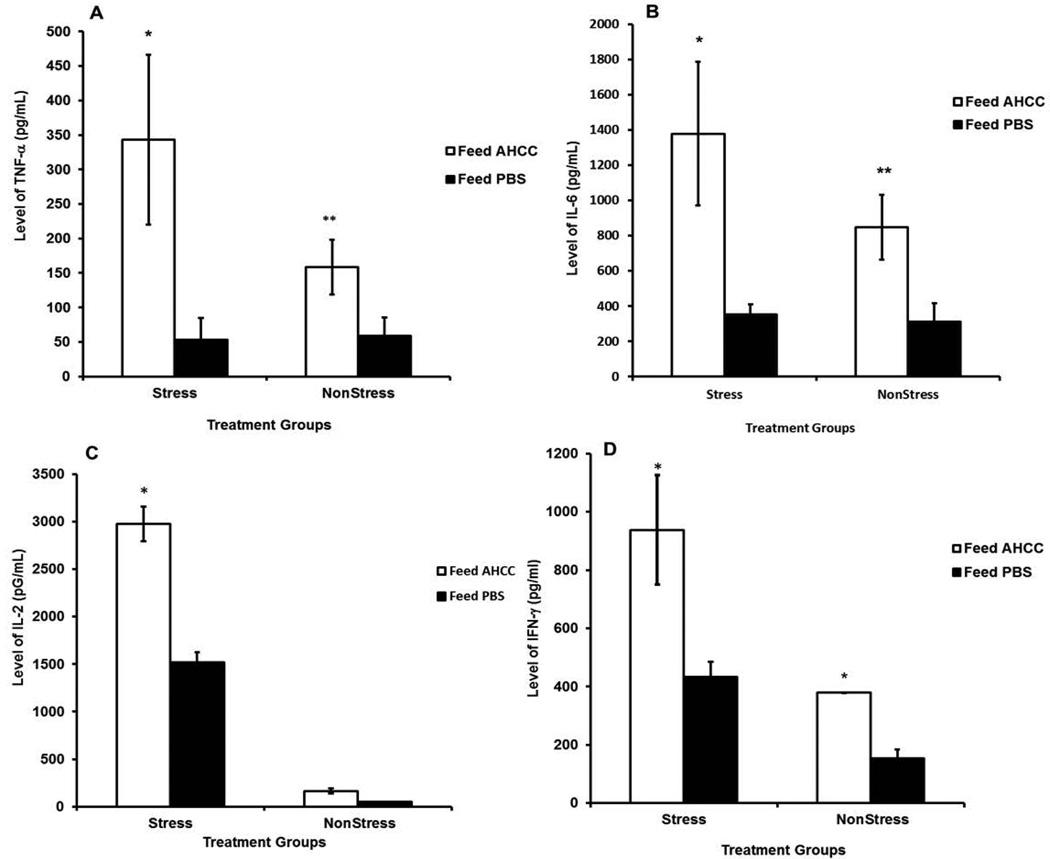
Effect of AHCC on the production of TNF-α (**A**) Interleukin-6 (**B**) by peritoneal cells stimulated with lipopolysaccharide; the production of interleukin-2 (IL-2) (**C**) and interferon-gamma (IFN-γ) (**D**) by spleen cells stimulated with Con A. Cytokine production in culture supernatants of in vitro proliferation cells was measured by ELISA. Data points are means ± SD of cytokine production in two separate experiments. * denote significant statistical differences between treatments groups (p < 0.05).

The function of splenic T cells and peritoneal cells isolated from AHCC-fed and PBS-fed mice was assessed for proliferation *in vitro* in the presence and absence of T cell mitogen concanavalin A (Con A) or peritoneal cell mitogen lipopolysaccharide (LPS), respectively. We measured the levels of key cytokines and results showed that production of cytokines, including TNF-α (Figure 1A) and interleukin-6 (IL-6) (Figure 1B) by peritoneal cells of AHCC-fed mice was significantly high compared to that of PBS-fed mice (*p* < 0.02). Similarly the production of IL-2 (Figure 1C), and interferon-gamma IFN-γ (Figure 1D) was significantly increased in splenic T cells of AHCC-fed mice compared PBS-fed mice. Moreover, production of TNF-α, IL-6, and IFN-γ in AHCC-fed stressed mice was higher than that of AHCC-fed non-stressed mice. Actually, AHCC feeding to non-stressed mice showed no change in IL-2 production over the control. Thus, the results suggested that AHCC could restore the cytokine production ability of stressed mice compared to non-stressed mice.

### Co-culturing of mouse monocyte J744.2 cell line with peritoneal cells enhanced TNF-α production

**Figure 2:**
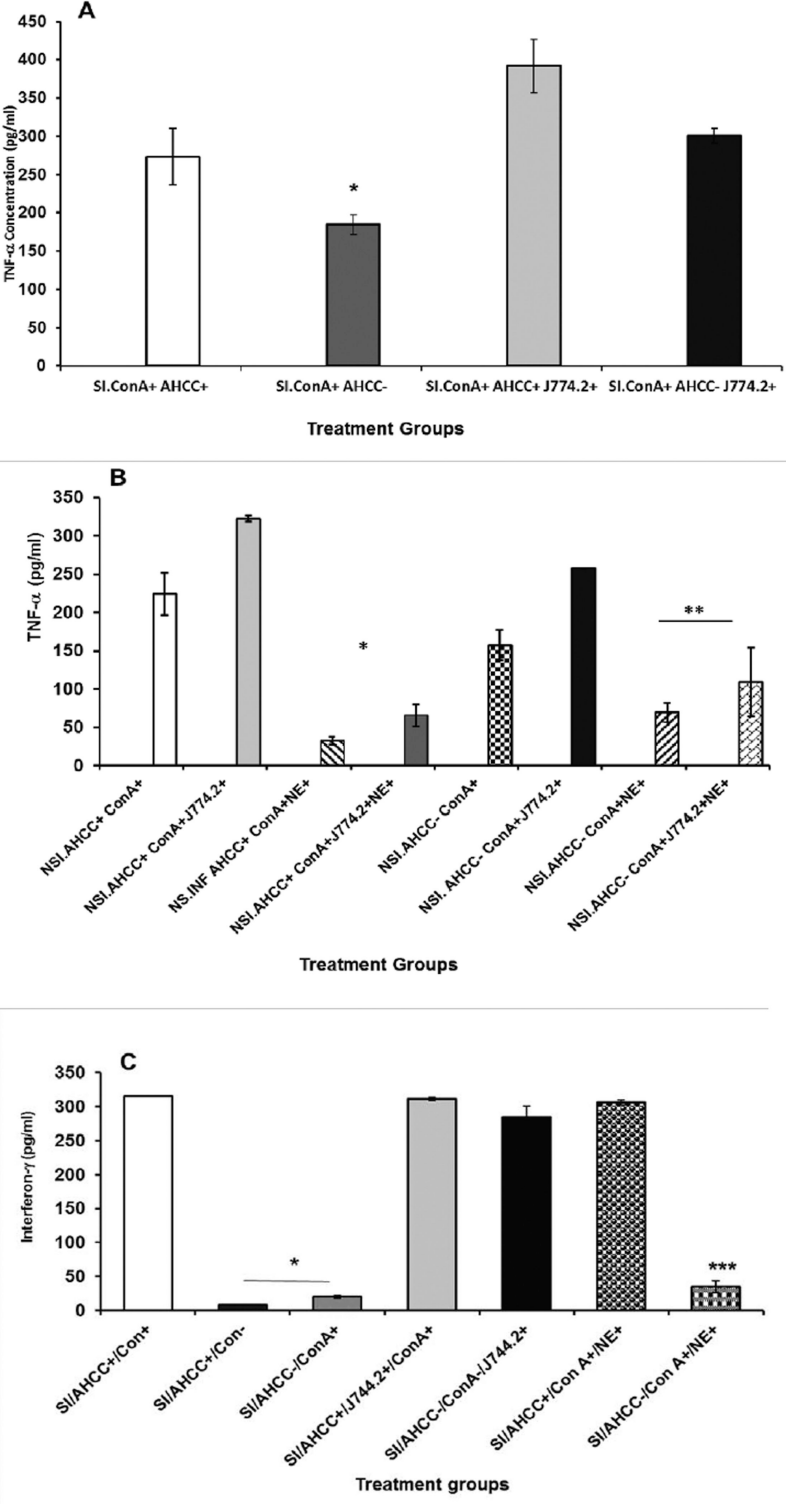
Effect of Active Hexose Correlated Compound-treated monocyte culture supernatants or Co-culturing with J744.2 cell line on the production of TNF-α by splenic T cells of stressed and chlamydia infected mice (SI) (**A**) or non-stressed and *Chlamydia trachomatis* infected (NSI) mice in the presence absence of Norepinephrine (**B**), or production of (interferon-gamma (IFN-γ) by splenic T cells of stressed infected mice (**C**) stimulated with Con A. See prev. pg. Data points are means ± SD at least duplicate well of two or more experiments.* denote significant statistical differences between treatments groups (p < 0.05).

We tested whether addition of AHCC to mouse monocyte, J744.2 cell line can lead to the production of interleukin-1β. The culture supernatants from AHCC-treated cell line were harvested after 24 h incubation, which had shown low detectable level of IL-1β by ELISA (**data not shown**). To assess the involvement of the culture supernatant of J744.2 containing IL-1β in stimulation of T cells, total splenic T cells harvested from stressed and non-stressed mice were co-cultured with J744.2 or cultured by adding a 100 µL of culture supernatant of AHCC-treated monocytes in the presence of ConA as mitogen. As shown in Figure 2A, co-culturing or addition of culture supernatant obtained from AHCC-treated mouse monocyte J744.2 to splenic T cells resulted in increased production of TNF-α in stressed mice compared to controls. Co-culturing of the J744.2 cell line with splenic T cells of stressed or non-stressed mice showed little or no difference to that of culture supernatant. In the presence or absence of AHCC, addition of culture supernatant of J744.2 to immune cells of non-stressed *Chlamydia trachomatis* infected mice resulted in increased production of TNF-α by splenic T cells, but less than that of the stressed mice. The results suggest that supplementation with AHCC to monocyte, J744.2 cell line increases the production of interleukin 1β, which subsequently stimulates the function of T cells as evidenced by the increased production of TNF-α. Addition of NE to cultures of immune cells in the presence of monocyte culture supernatant have resulted in a significantly diminished production of TNF-α (Figure 2B) indicating its inhibitory effect to the proliferation of immune cells and production of cytokines.

### Co-culturing of Splenic T cells with J744.2 or AHCC-treated monocyte culture supernatant enhanced INF-γ production

We tested the hypothesis that IL-β produced by AHCC-treated monocytes (J744.2) promotes increased production of INF-γ by total splenic T cells. Culturing of splenic T cells with culture supernatant obtained from AHCC-treated J744.2 resulted in increased production of INF-γ (Figure 2C). The data show that IL-β obtained from J744.2 is playing a key role in cytokine induction. It is speculated that interferon-γ is mainly produced by CD4^+^ T cells.

## Discussion

AHCC as a nutritional supplement is used widely world wide, but the basis of the advantages of nutritional supplements in the treatment and prevention of human diseases remains to be defined. The major objectives of this study were to test whether feeding AHCC to stressed mice decreases intensity of *Chlamydia trachomatis* genital infection and to elucidate the mechanisms of AHCC on the function of the immune cells in mice. Our data show that AHCC-fed, stressed mice had a substantial spleen weight gain compared to control groups. The weight gain of the spleen suggests that AHCC plays important role in redefining immune cell proliferation after damage during stressful conditions.

AHCC-feeding exhibited a reduced shedding of *Chlamydia trachomatis* from the genital tract associated with robust cytokine production indicating the recovery of immune cell functions. Furthermore, evidence from cytokine study shows that oral administration of AHCC to mice results in significantly increased cytokine production in stressed mice compared to non-stressed mice demonstrating the restoration of the functionality of immune cells. The relative increase in production of important cytokines (TNF-α, IL-6, IL-2 and INF-γ) in the AHCC-fed group may lead to decreased chlamydia genital infection in the stress model. Our results are similar to previous studies suggesting that AHCC affects the production of a variety of cytokines [31,34]. The results also demonstrated that AHCC-feeding of stressed mice resulted an overall reduction in the intensity of a primary *Chlamydia trachomatis* genital infection, but whether the robust production of cytokines have an essential role in the clearance of *Chlamydia trachomatis* remains to be determined. Based on these current findings we speculate that the damage of the immune system induced by cold-stress can increase susceptibility to *Chlamydia trachomatis*, but AHCC-feeding may restore the normal function of the immune system and minimize the propagation of the pathogen in the host. An overall increase of immune function in stressed mice equal or above control values suggests that AHCC has an impact on the immune function of stressed mice. In fact, AHCC restored the function of the immune system, which was suppressed by cold-induced conditions, to values very close to those obtained from mice under normal conditions. Therefore, whereas enhancement of the immune response in normal mice may have minimal beneficial effects on resistance to infection, restoration of the immune response to normal levels in immunosuppressed mice appears to be crucial for the control of immunopathogenesis.

The present observations and previous studies have led us to design mechanistic studies on the immunomodulation of AHCC treatment in our stress model. Previous studies have shown that AHCC treatment of monocytes results in the high production of IL-1β, which is essential for initiating and directing the development of immune responses by recruiting and providing activation signals to T cells [24,32]. We tested whether IL-β produced from AHCC-treated monocytes could promote an independently or dependently increase in the production of INF-γ by T cells (presumed to be more CD4^+^ cells) harvested from stressed and non-stressed mice. It was revealed that the exposure of T cells of stressed mice to culture supernatant of monocyte J744.2 produced more TNF-α compared to non-stressed mice, suggesting that the effect of AHCC is more significant in restoration of the function of damaged T cells than that of non-damaged T cells from non-stressed mice. Our data show that IL-β obtained from J744.2 may be playing a key role in inducing production as previously suggested [33]. It is speculated that TNF-α is cytokine produced by CD8^+^ T cells, and additional experiments are underway to determine which T cell subset is responsible for TNF-α production.

It has been shown that cold-induced stress alters the immune response [8,9] and compromises resistance to infection [7,13]. We have previously shown that AHCC can be used as a countermeasure to reduce the effects of hindlimb-unloading conditions [34]. However, the mechanisms by which AHCC increases immune function remains to be determined. Several studies in humans have shown the potential beneficial effects of AHCC on the immune system but very little is known about the effects of this compound on resistance to chlamydia genital infection. Our results are consistent with previous studies that show AHCC enhances the function of the immune system of humans [14–16,23–25,44] and rodents [19–21,25]. The enhancement of peritoneal macrophages by AHCC may result in activation of several components of innate immunity, including complement and acute phase proteins [45], inducing the synthesis and release of proinflammatory cytokines such as TNF-α, IL-1, and IL-6. This activation of the immune system could contribute to the rapid clearance of bacteria from the system, resulting in reduction of mortality in mice treated with AHCC. Innate and adaptive immunity appears to be greatly affected by cold-induced stress, but our data indicate that administration of AHCC may block the immunosuppressive effects of stress hormones. Additional *in vitro* studies are needed to clarify both the specific component(s) of AHCC that activates the immune system and the signaling pathways by which AHCC may be acting to upregulate the function of the immune system. In addition, several other factors must be considered in mice under cold-induced stress conditions that may have contributed to the immunological changes seen under stressful conditions. Factors involved may include those related to stress and hormonal changes, which may alter the immune system. Release of stress hormones such as corticosterones has been shown to inhibit immune responses and decrease resistance to infection [46–48]. AHCC is more effective in stressed mice suggesting that AHCC may be useful to protect against microbial infections in immunosuppressed hosts.

In summary, our data show that AHCC feeding to stressed mice results in (i) restoration of the functions of immune cells; (ii) reduced genital tract chlamydia shedding in AHCC-fed mice; and (iii) enhanced production of IFN-γ or TNF-α by T cell co-cultured with IL-1β producing monocytes. Since AHCC is believed to influence inflammation during stressful conditions by modulating the neuroendocrine stress response, we speculate that AHCC enhances immune function by multiple mechanisms, including enhancing key cytokine production in epithelial cells and macrophages that stimulate functions of T lymphocytes. Our ongoing study is characterizing the effect of AHCC on the signal transduction pathways of cytokine production. Defining the signal transduction pathways of AHCC immunostimulation and activation is a novel idea to prevent chlamydia genital infection. We will continue testing how AHCC treatment to cell lines or mice effects the protein profile IL-1 receptor-associated kinase (IRAK) family, tumor necrosis factor receptor-associated factor 6 (TRAF6), the kinase complex (I_κ_Bα), nuclear factor kappa B (NF_κ_B) by western blotting following manufacture instructions. We are testing *how* AHCC affects the level of stress hormone in our mouse model. Furthermore, we are interested in testing whether feeding stressed mice with AHCC results in reduced production of stress hormones, known to be suppressive to the immune system. The results of ongoing studies will provide important information in evaluating the immunotherapeutic potential of AHCC in targeting chlamydia genital infection in a mouse stress model. We can conclude that AHCC can be used as a potent immune enhancer, especially in cases in which the immune system is suppressed by any stressful conditions. It seems clear that AHCC reduces the level of *chlamydia trachomatis* genital infection and subsequently reduces of tissue injury and production of immunosuppressive cytokines. Furthermore, the results suggest that feeding AHCC to stressed mice promotes immune recovery that could have beneficial effects on the ability of the host to resist infection and thus our study will provide crucial data for further investigations in targeting approaches in treatment of Chlamydia genital infection in humans.

## Figures and Tables

**Table 1:** Effects of AHCC feeding on body and spleen weight of stressed mice.

Treatment Groups^a^	Initial Body Weight^b^	Body Weight At day 24 of stress	Weight Gain in %	Average Spleen Weight (g)
AHCC-fed Non-stressed	17.3±1.4	21.7±0.4	25.4%	0.1042 ± 0.01
AHCC-fed Stressed	17.4±0.5	21.1±0.3	21.6%	0.0963 ± 0.004
PBS-fed Non-stressed	18.46±1.6	20.96±0.6	13.5%	0.1495 ± 0.100
PBS-fed stressed	18.85±1.6	22.4±0.5	19.4%	0.0936 ± 0.11

a Treatment groups are stressed or non-stressed mice-receiving active hexose correlated compound or phosphate-buffered saline (PBS) by gavage for seven days before sacrifice.

b Body weight of each mouse in grams was measured every day during feeding. Mice were sacrificed seven days after feeding and weight of spleens was determined.

Statistical differences between treatment groups were compared at the level of (p < 0.05).

**Table 2:** Kinetics of *Chlamydia trachomatis* shedding from genital tract of mice after active hexose correlated compound feeding for 14 days^a^.

	Treatment groups			
Days after infection	Stressed, AHCC-fed	Stressed, PBS-fed	Non-stressed, no-fed	Non-stressed, AHCC-fed
Day 3	4.33±0.04	5.62±0.33	5.77±0.33	5.11±0.89
Day 18	1.19±0.01	3.46±0.76	3.21±0.90	1.00±0
Day 42	-	-	-	-

a values are mean +/− standard deviation of log_10_ inclusion forming unit/ milliliter representing combined results of two separate experiments (n = 5 to 6 mice per experiment).

No significant statistical difference between experimental groups was not observed on Day 3, but significant statistical differences between AHCC-fed and PBS-fed groups were obtained on Day 18.
